# Polysaccharide-Based Edible Films Incorporated with Essential Oil Nanoemulsions: Physico-Chemical, Mechanical Properties and Its Application in Food Preservation—A Review

**DOI:** 10.3390/foods11040555

**Published:** 2022-02-16

**Authors:** Ianne Kong, Pascal Degraeve, Liew Phing Pui

**Affiliations:** 1 Department of Food Science and Nutrition, Faculty of Applied Sciences, UCSI University, Jalan Menara Gading, UCSI Heights, Cheras, Kuala Lumpur 56000, Malaysia; 1001643671@ucsiuniversity.edu.my; 2 BioDyMIA Research Unit, Univ Lyon, Université Claude Bernard Lyon 1, ISARA Lyon, 155 rue Henri de Boissieu, F-01 000 Bourg en Bresse, France; pascal.degraeve@univ-lyon1.fr

**Keywords:** food preservation, essential oil, nanoemulsion, edible films, polysaccharide-based film

## Abstract

Edible films with essential oils (EOs) are becoming increasingly popular as an alternative to synthetic packaging due to their environmentally friendly properties and ability as carriers of active compounds. However, the required amounts of EOs to impart effective antimicrobial properties generally exceed the organoleptic acceptance levels. However, by nanoemulsifying EOs, it is possible to increase their antimicrobial activity while reducing the amount required. This review provides an overview of the physico-chemical and mechanical properties of polysaccharide-based edible films incorporated with EOs nanoemulsions and of their application to the preservation of different food types. By incorporating EOs nanoemulsions into the packaging matrix, these edible films can help to extend the shelf-life of food products while also improving the quality and safety of the food product during storage. It can be concluded that these edible films have the potential to be used in the food industry as a green, sustainable, and biodegradable method for perishable foods preservation.

## 1. Introduction

Foodborne illnesses caused by pathogens that threaten food safety are a major concern all over the world. According to the US Department of Agriculture, global food-borne illnesses cost more than US$15.6 billion each year. According to a World Health Organization (WHO) report issued in 2015, nearly 600 million individuals were affected by foodborne illnesses in 2015, with more than 420,000 deaths recorded worldwide [1]. Besides that, microbial contamination of food products has an impact on the economic growth of countries that rely on agricultural product exports, which contributes to the economic burden [2]. As a result, much recent research has focused on producing and utilizing bio-based polymers made from a variety of industrial by-products or food waste products, in order to work toward the development of environmentally friendly strategies for preventing microbial contamination of food products [3]. This increased interest is also due to the rising consumer demand for natural food preservation methods, which has led to the development of alternative protection methods, such as the use of edible biopolymer-based films or coatings derived from renewable sources or industrial by-products [4]. In this regard, in the last few decades, the development of innovative edible films and coatings has emerged as a new research area in food science.

Edible biopolymers and food-grade additives or ingredients are used to develop edible films and coatings. Polysaccharides (e.g., carbohydrates and gums) and proteins (e.g., gluten, casein, gelatin) are all examples of film-forming biopolymers [5]. Plasticizers such as glycerol and other additives, crosslinking agents, emulsifiers, and reinforcements or lipids (namely waxes) can be combined with the film-forming biopolymers to modify the physico-chemical, mechanical properties or the functionality of films. Edible films are thin layers of edible material that are formed as a protective coating or layer on foods and can be consumed alongside the food products [6]. They serve as a barrier between oxygen, moisture, and the surrounding environment. They can also be used to separate different compartments of the same food [7]. By limiting the exchanges between foods and their surrounding atmosphere, they can reduce water loss, oxidation reaction rates, and respiration rates, ultimately extending the shelf life of the food product [8]. Since most film-forming biopolymers are hydrophilic, their mechanical and physico-chemical properties can be altered by water. Therefore, decreasing the sensitivity of biopolymer-based films to water, in order to render possible their use in direct contact with rapidly perishable high moisture foods is a subject of intensive research. The crosslinking of biopolymers or the addition of hydrophobic compounds such as lipids in the formulation of films are common strategies which were successfully employed by several authors to decrease their sensitivity to water [9]. Furthermore, edible films may act as a carrier of active compounds such as antimicrobials, which can significantly improve the functionality of edible films in controlling or preventing microbial spoilage of food products by preventing microbial growth during the storage period, thereby not only extending the shelf-life of the food products but also maintaining their quality and safety [2,10].

Antimicrobials, antioxidants, colouring agents, flavours and nutraceuticals are among the active ingredients incorporated into film-forming suspensions to enhance the stability, quality and safety of packaged foods [11]. Interestingly, some of these ingredients may exhibit excellent antibacterial, antifungal, or antioxidant activity when incorporated into edible films and extend the shelf life and/or improve the safety of foods. Essential oils (EOs), aromatic oily liquids extracted from plant materials, are among the most common antimicrobial compounds incorporated in edible films [12]. Clove oil, lemon oil, cinnamon oil, tea tree oil, lavender oil, oregano oil, and peppermint oil are the most commonly used EOs in the production of active edible films or coatings. The key benefit of EOs is their antimicrobial activity against a wide range of microorganisms. Moreover, most of them have been approved by the United States Food and Drug Administration (US-FDA) as safe additives for food applications [2]. However, the amount of EOs used to impart effective antimicrobial activity may exceed organoleptic acceptance levels [13]. As a result, studies have proposed nanoemulsions as a new delivery system for encapsulating and releasing EOs from edible films into food products.

EO nanoemulsions have been used as active agents in the development of edible films by an increasing number of researchers during the last decade. The current review will include a discussion of the polysaccharides that have been investigated for the development of EO nanoemulsion-containing edible films. The various types of EO nanoemulsions that have been used in the development of edible films will be discussed. The mechanical and physico-chemical properties of edible films containing EO nanoemulsions will then be presented before giving examples of their application to the preservation of different types of foods.

## 2. Components of Edible Films

### 2.1. Polysaccharides and Their Properties

**Figure 1:**
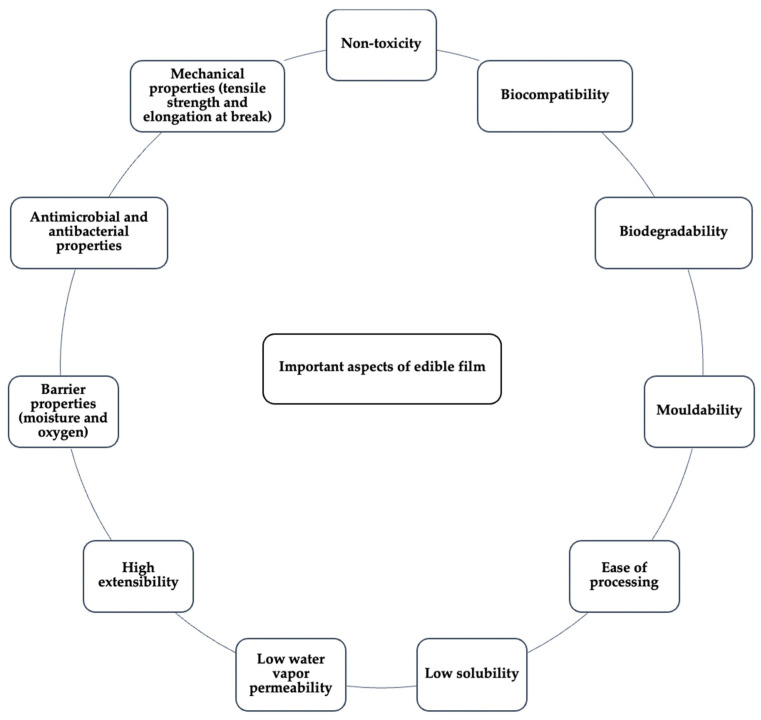
Important aspects of edible films.

Biopolymers, such as polysaccharides, that are derived from renewable sources are generally applied to the formulation of edible films. Polysaccharides have desirable properties for food-related applications, such as non-toxicity, good biocompatibility, biodegradability, ease of processing and mouldability [14,15]. As a result, polysaccharides have the potential to form films, making them particularly useful in food applications for food preservation. However, polysaccharide-based films have a few drawbacks. The disadvantages of these edible films are their high solubility in water, water vapor permeability, and low extensibility [16]. For instance, when polysaccharide-based films are applied to wrap meat products and exposed to smoking and steam, most of polysaccharide films dissolve and become integrated into the meat surface [17]. They also have poor moisture barrier properties and are thus unsuitable for liquid products due to their hydrophilic nature [17]. There are various important aspects to consider when developing active edible films in order to achieve the desired properties (Figure 1).

Nevertheless, polysaccharide-based films are extensively used in the food packaging industry to improve product shelf-life, stability, and safety, while using minimal packaging values that are invariably environmentally friendly due to the use of biodegradable materials. Furthermore, understanding the formulation of polysaccharide-based edible film-forming suspensions can aid in the preservation of physico-chemical properties and the shelf life of food products through the use of corresponding films or coatings [18]. Chitosan, pullulan, starch, alginate, carrageenan, cellulose, pectin, and gums are the main polysaccharides used in the preparation of edible films.

#### 2.1.1. Chitosan

Chitosan, a deacetylated derivative of chitin, is a linear amino polysaccharide containing d-glucosamine and *N*-acetyl-d-glucosamine units [19]. Chitosan has been used in the production of edible films due to its relatively broad spectrum antimicrobial activity [20], non-toxicity, biocompatibility, biodegradability, and solubility in acetic and hydrochloric acid, which results in film forming ability. The amount of chitosan used in the production of composite edible films affects both its physico-chemical and mechanical properties. When chitosan was added to corn starch-based edible films, it increased film solubility in water, total colour differences among the various edible film formulations, tensile strength up to 2.24 times that of the control, and elongation at break up to 3.24 times that of the control [21]. Chitosan can also incorporate additives and plasticizers like propylene glycol, glycerol, and polyethylene glycol.

Escárcega-Galaz et al. (2018) prepared [22] pure chitosan-based films by dissolving chitosan (1, 2, and 3% *w*/*v*) in 1% acetic acid with the addition of glycerol as plasticizer. The resulting edible films were smooth, homogeneous, transparent, and porous, with no fractures or cracks. The mechanical properties showed that the pure chitosan-based films are resistant to the breaking strength and that the addition of glycerol resulted in a higher percentage of elongation at break. These films also exhibited antimicrobial activity against *Klebsiella pneumoniae* and *Pseudomonas aeruginosa*. Furthermore, Abral et al. (2021) [23] also reported that all chitosan-containing films were effective against other Gram-negative bacteria (*Escherichia coli* and *Pseudomonas aeruginosa*) and also Gram-positive bacteria (*Staphylococcus aureus* and *Bacillus subtilis*) with inhibition zones of 12.8 mm, 15.1 mm, 20.9 mm, and 13.9 mm, respectively. The most commonly accepted mechanism of antimicrobial action of chitosan is based on the presence of positively charged amino groups of glucosamine and *N*-acetyl-d-glucosamine units along chitosan chains at acidic pH: these positive charges would favour the interaction of chitosan with negatively charged cell wall components of sensitive microorganisms and ultimately cause disruption of the cell. Once in the cell, the attachment of chitosan to DNA would cause inhibition of DNA replication and subsequently cell death [24]. Interestingly, Juliano et al. (2018) [25] also reported a synergistic antibacterial activity of EOs from Sardinian flora and sub-inhibitory concentrations of chitosan. Moreover, different EOs having a synergistic antimicrobial activity can be combined and added to chitosan-based films to limit the organoleptic defects often reported following the addition of single EOs at concentrations needed to effectively inhibit microbial food spoilage. This strategy was successfully used for inhibiting *E. coli* and *S. aureus* growth [26]. These authors also reported that *Origanum majorana* and *Ocimum basilicum* EOs mixture had an additive effect against both *E. coli* and *S. aureus* and that adding a mixture of *Origanum majorana* and *Thymus vulgaris* EOs in chitosan-gelatin films was the only combination of EOs active against these two bacterial species. The antimicrobial activity of Chitosan-based films can also be enhanced by adding other antimicrobial compounds in their formulation. For instance, chitosan-based edible film containing clove oil and nisin had shown possible synergistic antioxidative and antimicrobial activities by lowering the changes in metmyoglobin (MetMb) content, free fatty acids, peroxide value, and thiobarbituric acid reactive substances (TBARS) in pork patties [27]. In conclusion, the use of EOs combinations is a method of obtaining chitosan-based films with a broader antimicrobial activity spectrum.

#### 2.1.2. Starch

Starch is a promising natural polymer due to its abundance, annual renewability, inherent biodegradability, good mechanical properties, thermoplastic nature, and low cost. Starch is made up of a composition of amylopectin and amylose. Amylose is a sparsely branched carbohydrate mainly based on α-(1-4) bonds with a molecular weight of 10^5^ to 10^6^ anhydroglucose units, whereas amylopectin is a highly multiple-branched polymer with a high-molecular weight of 10^7^ to 10^9^ anhydroglucose units [28]. The chemical, physical and functional properties of starch-based films and coatings depend on the amylose and amylopectin ratio of each botanical source of starch [29]. Dai et al. (2019) [30] reported the effects of starches from different botanical sources (waxy corn (without amylose), cassava, sweet potato, potato, and wheat corn) and different commercial modified cassava starches (esterified cassava starch, cross-linked cassava starch, and oxidized cassava starch) on the physico-chemical properties of starch-based films. They noticed that waxy corn starch-based films had the highest water vapour permeability, while the cassava starch-based films had the lowest one due to the difference in amylose contents: waxy starch contains quite only amylopectin and no amylose, while cassava starch and wheat starch contain about 20% and 30% amylose, respectively. Furthermore, amylose and amylopectin contents, the size of starch granules in different botanical sources, and the physical and/or chemical treatments used for starch extraction are important factors conditioning starch properties. Wheat, corn, potato, rice, or cassava are common botanical sources of starch with known properties including film-forming ones. Some authors also tested the film-forming properties of starches extracted from less common botanical resources. For instance, Galindeza et al. (2019) [31] prepared edible films from the starch obtained from ulluco (*Ullucus tuberosus*). The ulluco starch-based edible films prepared demonstrated favourable mechanical properties, good stability against thermal degradation and low water vapour permeability, which is desirable to extend the shelf life of food products [31]. Arrowroot (*Maranta arundinacea*) starch was also used to prepare edible films [32]. Such films were homogeneous, transparent, and odourless, indicating that they have the potential to be used as a source to produce starch-based edible films for sustainable food packaging. Due to their hydrophilic properties, starch-based films generally have poor water vapour barrier properties but high oxygen barrier properties.

Several authors [2,12,13,33] reported that the incorporation of antimicrobial agents such as plant EOs into starch-based edible films reduces microbial growth and controls some enzymatic reactions in packed food products. However, most of EOs components are hydrophobic and thus have limited compatibility with the hydrophilic matrix of starch-based films. Therefore, EOs are generally emulsified before their addition in films formulation. Moreover, since perishable foods matrices are aqueous, emulsification of EOs will favour their delivery to perishable foods in direct contact and thereby their antimicrobial activity, as reviewed by Fu et al. (2016) [34]. As will be described in more detail later in this review, EOs nanoemulsions are colloidal dispersions of droplets having less than 500 nm diameter. Interestingly, this less than micrometric size of droplets confers to nanoemulsions a better kinetic stability compared to microemulsions. Moreover, it has been reported that the addition of lemongrass EO micro- or nano-emulsions in thermoplastic cassava starch-based films did not negatively affect their properties for food packaging applications, while conferring them with an antimicrobial activity [35].

#### 2.1.3. Pullulan

Pullulan, a linear extracellular polysaccharide, is synthesized in starch and sugar crops by the yeast-like fungus *Aureobasidium pullulans*. It is composed of α-1,6 glycoside linkages based on maltotriose [36,37]. Due to its non-toxic, biodegradable, water soluble, odourless, tasteless, and edible properties, it is a suitable polysaccharide for use in film production in the food industry fields [38]. Pullulan-based films are colourless, transparent, oil repellent, oxygen blocking, heat sealable, and can prevent the oxidation of oils and vitamins in food; however, the films have poor flexibility and are thus easy to break [39]. Nevertheless, due to the high cost of pure pullulan films and their poor properties, their use as edible films in food applications has been limited.

To address these issues, many studies have been performed to prepare composite films by incorporating pullulan with one or more other polysaccharides. In Chang et al. (2019) [40], β-glucan/pullulan composite edible films were successfully prepared with different ratios of pullulan and β-glucan by using the solvent casting method. The incorporation of β-glucan into pullulan-based edible films significantly increased the elongation at break, tensile strength, and water dissolution time of the resulting films, likely due to the formation of hydrogen bonds between pullulan and β-glucan. Furthermore, by preparing edible films with different pectin and pullulan proportions, the film’s surface became smoother and formed a denser structure, resulting in higher water vapour barrier properties [41]. Pullulan esters films were prepared from pullulan and different carboxylic anhydrides (acetic anhydride, propionic anhydride and butyric anhydride) [42]. When compared to pullulan-based films, pullulan esters-based films had better oxygen barrier properties. Strawberries packaged with pullulan esters-based films showed a significant reduction in weight loss percentage over time, retained firmness, and had a longer shelf life due to the films acting as semi-permeable barriers against oxygen, carbon dioxide, and moisture [42].

#### 2.1.4. Cellulose

Cellulose, a hydrophillic polymer, is the most abundant and renewable biopolymer in nature. Cellulose is made from a variety of raw materials, including wood, cotton, crops, cellulosic agriculture or food waste, and bacteria [43]. Chemical modification of naturally occurring cellulose can result in the formation of different forms of cellulose such as hydroxypropyl cellulose (HPC), carboxymethyl cellulose (CMC), hydroxypropyl methyl cellulose (HPMC), methyl cellulose (MC), or ethylcellulose (EC) [44]. Due to their biodegradability, excellent mechanical properties, and sustainability, these five types of cellulose derivatives are commonly used in the production of edible films and coatings [23].

Abral et al. (2019) [45] utilized different chemicals and ultrasonication to produce a transparent cellulose film from ginger nanofiber. The cellulose film produced demonstrated high thermal stability, with a maximum decomposition temperature peak at 353 °C, as well as good antimicrobial properties due to bioactive compounds in ginger fibers that inhibit the growth of bacteria. Besides that, cellulose films were successfully produced from durian rind in the study carried out by Zhao et al. (2019) [43]. Tensile tests revealed that the cellulose film exhibited high rigidity and tensile strength, as well as a good appearance with a smooth surface and excellent transparency. The biodegradation analysis showed that the film could be completely broken down in soil in four weeks, with a higher biodegradation percentage than cellophane, indicating that the film has excellent biodegradability.

#### 2.1.5. Gums

Hydrophobic or hydrophilic plant-based gums, for example, can be found in plants and woods, where they are derived from plant cell walls, tree exudates, seed endosperm, and tubers. The types of commonly used gums to produce edible films are seed gums (guar gum, locust bean gum), microbial fermentation gums (xanthan gum, gellan gum) and exudate gum (arabic gum, ghatti gum, karaya gum, tragacanth gum) [46]. They are widely used in edible film formation due to their texturizing proficiency [46]. Different combinations of xanthan and locust bean gum were used in the preparation of biodegradable edible films [23]; interestingly, these authors [23] reported that the exploitation of synergistic interaction between locust bean gum and xanthan resulted in films with improved properties and good miscibility. In another study [47], different ratios of psyllium gum and modified starch (100:0, 50:50, 75:25, 0:100) were used to produce edible films. Fourier transform infrared (FTIR) spectra of the films demonstrated miscibility and good interactions between modified starch and psyllium gum. Moreover, the elongation at break (EB) of psyllium gum-modified starch edible films increased when the gum content exceeded 50%. Other authors [48] developed and characterized an *Alyssum homolocarpum* seed gum-based biodegradable edible film. The functional properties of *Alyssum homolocarpum* seed gum-based films were found to be comparable to those of common biopolymer-based films such as starch- or gelatin-based films.

#### 2.1.6. Pectin

Pectin is the soluble component of plant fiber derived from fruits and vegetables. It is a heterogeneous group of acidic structural polysaccharide linkage with β-1,4-linked d-galacturonic acid residues [44]. High Methoxyl pectin (HMP) or Low Methoxyl pectin (LMP) are characterized by different levels of methyl esterification of the carboxyl groups of galacturonic acid residues [49]. Pectins can be extracted from fruit pomaces, which are by-products of fruit juice production. In this regard, they are thus considered as a sustainable resource for edible films production. Pure pectin films are homogenous and clear, however, such films have poor moisture barrier properties [50].

Interestingly, Chaichi et al. (2017) [51] reported that reinforcement by 5% of crystalline nanocelullose (CNC) of pectin-based films resulted in an increase of up to 84% in tensile strength and in a decrease in water vapor permeability of up to 40%. Chen et al. (2020) [52] also observed that LMP-based films were strengthened by incorporating ellagitannins from the unripe fruits of *Rubus chingii* Hu and tara gum: their addition increased thickness and water resistance of films.

#### 2.1.7. Alginate

Alginates are structural polysaccharides extracted from *Phaeophyceae* seaweeds (brown algae) like *Macrocystis*, *Laminaria*, and *Ascophyllum* [53]. Alginate, a natural linear and anionic polysaccharide, is an attractive biopolymer for its favourable properties, such as low toxicity and chemical versatility [19]. Alginate has advantageous properties in the production of edible films due to its unique colloidal properties, which include thickening, suspending, stabilizing, film-forming, emulsion stabilizing, and gel-producing properties in the presence of calcium ions [53].

The alginate-based edible films are resistant to oil and grease [44]. One of their appealing properties is the ability to improve food quality and shelf life by reducing dehydration and controlling respiration of fruits and vegetables [53]. Alginate-based edible films provide a good oxygen barrier, which helps to delay lipid oxidation in vegetables and fruits and reduce weight loss and the abundance of microflora on the surface [54]. Based on Reyes-Avalos et al. (2019) [55], the use of an alginate-chitosan film on fresh figs (*Ficus carica*) successfully delayed the ripening process by reducing O_2_ and increasing CO_2_ concentrations. Furthermore, the alginate-chitosan film also successfully preserved the total polyphenol content of the figs, as well as the identified individual polyphenols.

#### 2.1.8. Carrageenan

Carrageenans are one of the major galactan groups found in red seaweeds [56]. The most commonly used seaweeds for carrageenan extraction are *Kappaphycus alvarezii* and *Eucheuma denticulatum* [57]. Based on the distribution of 3,6-anhydro-d-galactose residues and their sulphation patterns, carrageenans are classified into several sub-types [58]. Carrageenans are widely used as gelling, emulsifying, and stabilizing agents in the food, dairy, and pharmaceutical industries [59]. Besides that, because of its excellent gel and film-forming properties, carrageenan is frequently used in the formation of edible films [60].

Praseptiangga et al. (2016) [61] produced semi-refined-kappa-carrageenan-based edible films and carrageenan-based edible films containing cinnamon EO. The thickness of film and elongation at break were enhanced by the increase in sorbitol concentration levels, sorbitol being used as a plasticizer.

#### 2.1.9. Mucilage

Mucilage is a polymeric gel produced by most plant roots and consists primarily of carbohydrates, amino acids, and organic acids, with a minor amount of glycolipids and other phospholipids [62]. Furthermore, they are physiological intracellular formation products in plant metabolism, retaining a large amount of water in vegetables [63]. They are classified as heteropolysaccharides and are a potential alternative for the production of edible films.

In recent years, research on mucilage-based films developed for edible purposes using various plant types has been conducted. These mucilage-based films have also been incorporated with EOs in order to investigate the properties of the film produced. Behbahani et al. (2020) [64] has incorporated cumin essential oil (CEO) with Shahri Balangu seed mucilage (SBM) to develop an effective and environmental edible coating for beef slice. The edible film produced yielded positive results in that it improved the quality and microbial safety of beef slices by reducing microbial populations and lipid oxidation.

### 2.2. Edible Films Incorporated with Essential Oil Nanoemulsion

Edible films can be formulated from different materials and food grade chemicals to obtain desired properties. When edible films are incorporated with certain additives into packaging films (e.g., induction of the inner side of films in contact with food with edible coatings) or within packaging containers with the goal of maintaining and extending product shelf-life, this is referred to as active packaging [39]. Active packaging is a novel concept in the field of food science that can benefit fresh foods by extending their shelf life and by improving the quality of food products. According to Article 3 of the European Parliament and Council (EC) No. 1935/2004 regulation, all materials and articles in active packaging intended for contact with food must be safe [65]. However, in the European Union, active edible packaging is subject to a number of regulations. Likewise, materials or articles in active packaging that are intended to come into contact with food are regulated by Regulations No. 1935/2004 and its amendments, as well as Regulation No. 2023/2006 [66]. Active packaging must be sufficiently inert to prevent their constituents from transferring into food in quantities that could threaten human health, or that cause undesirable changes in the characteristics of the food or deterioration of the food’s organoleptic qualities under regular and inevitable use conditions [66,67].

The Commission Regulation (EC) No. 450/2009 on active and intelligent materials and articles intended to come into contact with food contains detailed regulations on the use of active packaging [67]. Active materials and articles must be purposefully formulated so that the substances in them either release or absorb substances into the packed food or the environment, and they must be subjected to a safety assessment by the European Food Safety Authority [67]. The substances released from active materials must meet the requirements in such a way that, based on the scientific evidence available, it does not pose a safety concern to the consumer’s health at the level of use proposed [68].

Antioxidant and antimicrobial agents are the most frequently used active additives or ingredients incorporated in edible films to prevent bacterial growth, improve safety and/or shelf life, and increase consumer confidence.

Natural food ingredients such as plant EOs are generally used as flavouring agents in food products [69]. EOs could be helpful for application of edible film in the food packaging industry due to their antimicrobial and antioxidant properties [70]. EOs inhibit microbial growth by interfering with the cytoplasmic membranes, destroying cell constituents, and reacting with cell membranes, leading to increased permeability, cell component loss, and coagulation of cell contents [71]. Numerous studies have been conducted to demonstrate the antimicrobial properties of EOs against a wide range of food spoilage and pathogenic microorganisms [72]. However, the required amounts of EOs to impart effective antimicrobial properties generally exceed the organoleptic acceptance levels [73]. Besides that, EOs are poorly soluble in water and can denature when exposed to light and heat [69]. Therefore, there is a need to investigate new delivery systems to encapsulate and release EOs in food products. Since EOs are rather hydrophobic, while edible film-forming polysaccharides are rather hydrophilic, dispersing EOs in emulsions is a way to improve the compatibility between EOs and polysaccharides before their incorporation in the formulation of film-forming suspensions.

Nanoemulsions are emulsions with very small droplet sizes, typically less than 100 nm [73]. There are several methods for producing stable nanoemulsions. The formation of nanoemulsions can be accomplished in two ways. The first is through high-energy homogenization using specialized mechanical devices such as high-shear mixers, high-pressure homogenizers, sonicators, or micro-fluidizers. The second method is the low-energy method, which includes spontaneous emulsification, phase-inversion temperature, phase-inversion point, and phase-inversion composition [74]. As a new delivery system, nanoemulsions have two important properties for encapsulating and releasing EOs from edible films into food products. Nanoemulsification has improved the physicochemical stability of EOs as well as their biological properties by increasing the specific surface area and thus lowering the amount of active compounds required [75]. Because nanoemulsions can increase solubility, bioaccessibility, and bioavailability, while also maintaining stability of EOs, they are suitable for incorporation into edible film as an active food coating or packaging. The EO nanoemulsions-based edible films have thus the potential to improve the quality of a variety of food products by extending their shelf life.

#### 2.2.1. Stability of Essential Oil Emulsion and Nanoemulsion

Droplet size and polydispersity index (PDI) are important parameters for determining emulsion stability [76]. In general, the smaller the particle size, the better the kinetic stability, solubility, and functionality as a carrier of the emulsion or nanoemulsion [77]. PDI values less than 0.25 imply a narrow size distribution of particles with good stability [78]. The stability of an emulsion is usually determined by keeping it at various temperatures for an extended period of time. Therefore, Xiong et al. (2020) [79] stored oregano EO emulsions and nanoemulsions at 4 and 25 °C for 15 days to test their stability. All of the PDI values in this study were less than 0.25, indicating that both emulsions and nanoemulsions were stable during the 15-day storage period. while the particle size of all groups increased gradually over time, oregano EO nanoemulsions maintained smaller particle sizes than standard emulsions after 15 days at both temperatures (4 °C and 25 °C). This clearly indicates that nanoemulsions outperform emulsions in terms of storage stability.

Chu et al. (2020) [80], on the other hand, compared the particle size and PDI of pullulan-based coating solutions incorporated with cinnamon EO nanoemulsion to coarse emulsion. The results showed that pullulan-based coating solutions incorporated with cinnamon EO nanoemulsion had significantly smaller droplet size (162.1 nm) than coarse emulsion (210.6 nm). Besides that, cinnamon EO nanoemulsion had a lower PDI (0.193) than coarse emulsion (0.393). Similarly, Shokri et al. (2020) [81] found that the particle size of *Ferulago angulata* EO nanoemulsion was 99.5 nm in chitosan-based coating solutions, which was smaller than the coarse emulsion, which had a droplet size of 462 nm. The *Ferulago angulata* EO nanoemulsion had a significantly lower PDI than the coarse emulsion, with PDI values of 0.26 and 0.53, respectively. It was concluded that after nanoemulsification, the average droplet size of coarse emulsions was dramatically reduced, which was accompanied by a significantly more positive ζ-potential and PDI, which thereby stabilized the EO. According to these two studies, nanoemulsifying EOs helps to increase their stability by reducing particle size and PDI.

#### 2.2.2. Film Formulation

Consumers’ growing concern about the safety of some synthetic chemical preservatives (e.g., sulphites, nitrites) has resulted in an increase in the use of EOs as antioxidant and antimicrobial agents in the food industry [82]. As antimicrobial agents, nanoemulsions based on polysaccharides such as starch and EOs could be used to produce active edible films, ushering in a new era of edible packaging. Numerous studies have been conducted on the incorporation of nanoemulsified EOs into edible films as active packaging in order to improve both the quality and shelf life of a food product. To create an active edible film with improved properties, various food grade ingredients, additives and processing aids were used as a plasticizer or a solvent, with the addition of nanoemulsified EOs to formulate the film forming suspensions. Table 1 lists examples of formulations of different polysaccharide-based edible films incorporating various EO nanoemulsions.

Acevedo-Fani et al. (2015) [10] produced alginate-based edible films by incorporating the nanoemulsion of EOs from various plants such as thyme, lemongrass, and sage to improve their dispersion in water and protect EOs from degradation. The nanoemulsified EOs were prepared by microfluidization treatment at a temperature below 15 °C. The alginate-based edible films were prepared by dissolving sodium alginate in distilled water at 70 °C, followed by the mixing of glycerol (2% *v*/*v*), Tween^®^ 80 (3% *v*/*v*) and thyme, lemongrass, or sage EO (1% *v*/*v*) in a high speed blender at 17,500 rpm for 2 min at room temperature. Moghimi et al. (2017) [75] prepared a *Thymus daenensis* EO nanoemulsion by using a probe sonicator with an amplitude of 30% for 15 min at a constant temperature to prepare hydroxyl propyl methyl cellulose (HPMC) films with EO nanoemulsion. Different kinds of polyethylene glycol (PEG) (300, 400 or 6000) were used as plasticizers in this study. In the Hashemi et al. (2013) study [13], nanoemulsion of *Zataria multiflora* EO was prepared and incorporated into basil seed gum-based films. The aim was to increase the bioactivity of *Zataria multiflora* EO by making a nanoemulsion. *Zataria multiflora* EO nanoemulsion was produced using high intensity ultrasound at 150 W and different sonication times (0, 2.5, 5 and 10 min).

Aisyah et al. (2018) [82] formulated corn starch-based edible films incorporating various concentrations of nutmeg EO nanoemulsion (1%, 2%, and 3% (*v*/*v*)) and glycerol (10%, 20%, and 30% *v*/*v*). To determine the suitability of using nutmeg EO nanoemulsion in developing the film matrix, they evaluated the mechanical properties and antibacterial activity of corn starch-based edible films. The study found that a 1% nutmeg oil nanoemulsion could inhibit the growth of two bacteria tested (*Staphylococcus aureus* and *Escherichia coli*) films. Xiong et al. (2020) [79] evaluated the effect of oregano EO and resveratrol nanoemulsion loaded pectin edible film on fresh pork loin preservation under high oxygen modified atmosphere packaging. The prepared oregano EO and resveratrol nanoemulsion showed good stability at 4 °C for 15 days. The pectin edible film was formed by dissolving citrus peel pectin in water and adding 0.4% (*v*/*v*) ethanol, 1.25% (*v*/*v*) Tween^®^ 80 with the addition of 0.5% (*v*/*v*) oregano EO, and 200 mg·L^−1^ resveratrol [79]. Shokri et al. (2020) [81] carried out nanoemulsification of *Ferulago angulata* EO in order to improve the efficiency of chitosan-*Ferulago angulata* EO edible film in extending the shelf life of rainbow trout fillets. Acetic acid was used as the solvent for the film forming suspensions, which included *Ferulago angulata* EO nanoemulsion at 1, 2, and 3% (*v*/*v*) concentrations that had previously been thoroughly mixed with Tween^®^ 80.

Chu et al. (2020) [80] developed pullulan-based films enriched with cinnamon EO nanoemulsion prepared using ultrasound. Pullulan, water, glycerol as a plasticizer, non-ionic surfactant Tween^®^ 80, and 8% (*v*/*v*) cinnamon EO nanoemulsion were added in the formulation of films. The storage effect of the pullulan-cinnamon EO nanoemulsion films on fresh strawberries during room storage was investigated. When compared to untreated strawberries and strawberries coated with pure pullulan-based films, strawberries coated with pullulan-based films enriched with cinnamon EO nanoemulsion had a four day longer shelf life. Cinnamon EO nanoemulsion was also incorporated into a bioactive film made of soluble soybean polysaccharide (SSPS) [83]. SSPS film-forming solutions (3% *w*/*v*) were prepared with the addition of glycerol and cinnamon EO nanoemulsion in varying concentrations (0, 0.2, 0.4, 0.6, and 0.8% (*v*/*v*)). In order to investigate its applicability to perishable food preservation, the SSPS-cinnamon EO nanoemulsion film was applied on meat during its refrigerated storage for 8 days. A cinnamon EO nanoemulsion was prepared by Frank et al. (2018) [84] using a probe-type ultrasonication apparatus to produce alginate-based antibacterial biocomposite films. The alginate-cinnamon EO nanoemulsion films were then prepared by incorporating the cinnamon EO nanoemulsion into an aqueous suspension of alginate and glycerol, homogenizing, casting, and subsequent drying.

Restrepo et al. (2018) [72] studied the mechanical, barrier, and physico-chemical properties of banana starch edible films incorporated with nanoemulsions of lemongrass (*Cymbopogon citratus*) and rosemary (*Rosmarinus officinalis*) EOs. The emulsion-phase inversion method was used to produce lemongrass and rosemary EO nanoemulsions, which were then cast to form edible films with banana starch and glycerol. Based on Gharibzahedi and Mohammadnabi (2017) [85], nettle EO nanoemulsion was incorporated into jujube gum-based edible films. To prepare the jujube gum-nettle EO nanoemulsion films, the nettle EO nanoemulsion was homogenized in an aqueous phase containing Tween^®^ 40 surfactant, glycerol as a plasticizer, and jujube gum dispersed in acetate buffer. The jujube gum-nettle EO nanoemulsion film was tested on Beluga sturgeon fillets. Dini et al. (2020) [86] successfully developed a chitosan film containing a nanoemulsion of cumin EO to evaluate the edible films effect on the microbiological safety and quality of beef loins during chilled storage. The edible chitosan-cumin EO nanoemulsion film was produced by dissolving chitosan in 1% (*v*/*v*) glacial acetic acid with the addition of glycerol as a plasticizer, then adding cumin EO nanoemulsion in Tween^®^ 80 and, lastly, homogenizing and drying the suspension.

Almasi et al. (2019) [87] activated pectin films by incorporating marjoram EO nanoemulsion and pickering emulsions. Tween^®^ 80, a low molecular weight surfactant, and a blend of whey protein isolate and inulin were used to make nanoemulsions and pickering emulsions, respectively. The materials used to prepare pectin films were citrus pectin, water, glycerol, marjoram EO-loaded nanoemulsion and pickering emulsions dispersions. Hossain et al. (2018) [88] used a three-level full factorial experimental design to optimize the microfluidization pressure to develop methyl cellulose (MC)/cellulose nanocrystal CNC-based nanocomposite films containing a blend of thyme and oregano EOs nanoemulsion [88]. Blended thyme and oregano EOs were prepared using lecithin and Tween^®^ 80 as surfactants, followed by film formation with the solubilization of MC and CNC in distilled water and the addition of glycerol as a plasticizer. The desired amount of CNC, MC, and EO emulsions were then homogenized at room temperature and stored in a beaker before further homogenization or microfluidization. To extend the shelf life of fresh-cut oranges, Radi et al. (2017) [89] incorporated orange peel EO nanoemulsion in a pectin-based film. The nanoemulsion of orange peel EO was prepared using Tween^®^ 80 as a surfactant. To prepare the active pectin-based film, pectin was dispersed in double distilled water before being mixed with orange peel EO nanoemulsion. The mixture was then homogenized using a high shear homogenizer.

## 3. Characterization of Essential Oil Nanoemulsion Loaded Edible Films

Recent research has shown that EOs-loaded nanoemulsions have improved the physical and mechanical properties of films when compared to the effect of incorporation of conventional emulsions. Furthermore, higher antibacterial activity has also been observed in nanoemulsified EOs [80]. For instance, pullulan-cinammon EO nanoemulsion coated strawberries had the highest inhibitory activity against bacteria and moulds at the end of the storage period, with log CFU/g values of 2.544 and 1.958, respectively, whereas the pure pullulan coated samples all had values that exceeded 10^3^ (bacteria) and 10^4^ (moulds) CFU/g [80]. In this regard, polysaccharide-based edible films such as pectin and alginate could be incorporated with EO nanoemulsions as antimicrobial agents during the manufacturing process, resulting in a new generation of active edible coatings/packagings.

It is necessary to evaluate the wettability, the physical, chemical as well as the mechanical (tensile strength, elongation at break), thermal, optical (brightness, opacity), and morphological properties of edible films incorporated with nanoemulsified EOs. This is necessary since the EO type and its interaction with the matrix determine the effectiveness of the edible films as food packaging materials [90]. Moreover, edible films also influence the movement of gases and act as a barrier to aromatic compounds transfer; they can thus also be used to create a modified atmosphere [91]. In this context, it is necessary to evaluate whether the gas barrier properties of edible films are affected by the nanoemulsified EOs addition in their formulation. Furthermore, when edible films are applied to food products for storage, their properties may influence consumer acceptability and their industrial applicability [90]. As a result, it is essential to consider the physico-chemical and mechanical properties of edible films when they are combined with EO nanoemulsions.

### 3.1. Physical Properties

#### 3.1.1. Colour

Colour is one of the optical properties of edible films that can improve or degrade the overall appearance of food products, thereby influencing consumer acceptance. Colour can be measured by using a L*, a*, b* colorimeter [10,72]. Acevedo-Fani et al. (2015) [10] reported that alginate-based films containing thyme EO nanoemulsions had the highest positive value (6.9 ± 0.6) expressed by coordinate b*, indicating that these films had a light greenish-yellowish tone. This might be due to the presence of phenolic compounds in thyme EO, which may have low wavelength light absorption [92]. Similarly, the addition of cinnamon EO nanoemulsions into edible films increased the yellowness (b*), indicating that increasing the concentration of cinnamon EO nanoemulsions caused the colour of the alginate bio-composite films to become slightly yellowish in colour [84].

Lightness (L*), redness (a*), and whiteness were significantly reduced by increasing the concentration of cinnamon EO nanoemulsions in soluble soybean polysaccharide-based edible films, according to Ghani et al. (2018) [83]. Adding cinnamon EO nanoemulsions increased yellowness (b*), as in alginate films. The results revealed that increasing the concentration of cinnamon EO nanoemulsions changed the colour of soluble soybean polysaccharide-based edible films from light yellow to red. Unlike with cinnamon EO, Restrepo et al. (2018) [72] reported that increasing the concentration of rosemary and lemongrass EO nanoemulsions did not affect the L*, a*, and b* values of banana starch edible films.

#### 3.1.2. Film Thickness

Thickness is an important parameter when studying the mechanical and water vapour barrier properties of films [87]. The film thickness was found to have a significant relationship with the droplet size of EO nanoemulsions [10]. Aisyah et al. (2018) [82] reported that corn starch-based edible films with 3% (*v*/*v*) nutmeg EO nanoemulsion had the highest thickness, while the incorporation of a 1% (*v*/*v*) nutmeg EO nanoemulsion resulted in the lowest thickness. The results show that the higher the concentration of nutmeg EO, the thicker the edible films produced. Consistently, Moghimi et al. (2017) [75] observed that hydroxyl propyl methyl cellulose (HPMC) films incorporating the *Thymus daenensis* EO nanoemulsion were significantly thicker than the control films that did not contain nanoemulsions.

The components of EO nanoemulsion, which consist of EO, water, and surfactant, may be responsible for the increase in the thickness of edible film. When the volume of solution poured on each plate is the same, the difference in thickness is caused by differences in the concentration of total solids in the film-forming suspensions [82]. The increase of the viscosity of the film-forming suspensions following the addition of EOs nanoemulsions could also result in thicker edible films. Indeed, Bertuzzi et al. (2007) [93] also demonstrated that increasing the glycerol concentration increased the viscosity of the solution, thereby increasing the film thickness.

The thickness of basil seed gum-based edible films incorporated with *Zataria multiflora* EO nanoemulsion ranged from 114 to 130 μm [13]. There was thus no significant difference between the thickness of basil seed gum control films and of those incorporated with *Zataria multiflora* EO nanoemulsion. Acevedo-Fani et al. (2015) [10] even reported that alginate-based films containing thyme EO and sage EO nanoemulsions presented a significantly smaller thickness compared to control films. Furthermore, incorporation of 2.5 and 5% (*v*/*v*) marjoram EO nanoemulsion significantly decreased the thickness of pectin-based edible films [87]. Previous research suggested that thickness reduction in edible films could be caused by emulsions with small droplet sizes. This effect was attributed to possible EO phase losses during the formation of films, which could reduce the total amount of solids concentration in the film matrix [10].

#### 3.1.3. Film Solubility in Water

Film solubility in water is one of the most important criteria for selecting a film for a specific application. As can be seen in Ghani et al. (2018) [83], the solubility of soluble soybean polysaccharide-based edible films decreased with the increasing content of cinnamon EO nanoemulsions. Furthermore, when banana starch edible films were combined with nanoemulsions of lemongrass and rosemary EOs, the film solubility at 25 °C and 95 °C of the films decreased [72]. The findings obtained in both studies could be explained by the inclusion of hydrophobic compounds that do not solubilize in water, thus maintaining the edible film’s structure in water [83].

Hashemi et al. (2017) [13] observed that the solubility of basil seed gum-based edible films in water was reduced when the edible films were incorporated with *Zataria multiflora* EO nanoemulsion. It could be attributed to the increased hydrophobicity of *Zataria multiflora* EO-loaded basil seed gum films, as well as to network strengthening as also discussed for mechanical properties [83]. Network strengthening can be caused by extensive hydrogen bondings (dipole-dipole interaction) between polar groups, particularly hydroxyl groups, present along the polymer backbone and polar head groups of surfactant molecules present at the interface of dispersed nanodroplets [13].

### 3.2. Physico-Chemical Properties

#### 3.2.1. Water Vapour Permeability

Water vapour permeability (WVP) is one of the most important factors to consider when selecting a packaging for food storage [83]. This parameter can be used to estimate the barrier property of a film by measuring the diffusion of water molecules through its cross-section [10]. Films used as packaging or coatings must control moisture transport from the product to the environment to prevent or reduce food dehydration, therefore, the WVP of edible films should be as low as possible [94].

EOs are known to decrease the WVP of polysaccharide-based films due to their hydrophobic properties [10]. Incorporation of 0.2% (*v*/*v*) of cinnamon EO nanoemulsion into soluble soybean polysaccharides-based edible films had no significant influence on WVP when compared to the control films. However, when the cinnamon EO nanoemulsion concentration was increased from 0.4 to 0.8% (*v*/*v*), the WVP of soluble soybean polysaccharides-based edible films was significantly lowered when compared to the control films [83]. Increasing the concentration of EOs nanoemulsion up to a certain threshold concentration reduced the WVP of films, most likely because the inclusion of nanoemulsion increased the hydrophobic character of the film solutions [10]. Above this threshold concentration of EO nanoemulsion, the high contact between soluble soybean polysaccharides and EO nanoemulsion droplets lowers the soluble soybean polysaccharides chain aggregation forces, thereby reducing the conjunction forces of the polymer network [83].

Similarly, Acevedo-Fani et al. (2015) [10] also observed a significant reduction of WVP when sage EO nanoemulsion was incorporated into alginate-based edible films. This could be explained by the corresponding nanoemulsions’ small droplet size, which leads to a more even dispersion of the oily phase in the film structure. Nanocomposites containing well-dispersed nanoemulsions up to an “optimum” proportion have been observed to have improved barrier characteristics [95]. Furthermore, when methyl cellulose (MC)/cellulose nanocrystal (CNC)-based nanocomposite films containing a blend of thyme and oregano EOs nanoemulsion were compared to MC-based control film in Hossain et al. (2018) [88], the WVP values reduced by 9%. This significant decrease in WVP was attributable to CNC dispersion in the matrix, which resulted in a longer path length for water vapour diffusing molecules due to the tortuosity effect.

#### 3.2.2. Moisture Absorption

Most food products contain a significant amount of water. Variations in moisture content can cause major changes in food stability and quality [96]. As a result, moisture absorption is another aspect to consider when looking into the water barrier qualities of film samples. The weight loss observed by the bio-composite films after 24 h of oven drying until a consistent dry weight was reached can be used to measure their moisture content [84].

The percentage of moisture absorption of pectin-based edible film was significantly increased after the addition of 2.5 and 7.5% (*v*/*v*) marjoram EO nanoemulsion in pectin-based edible films [87]. The control and alginate-based edible films with high cinnamon EO nanoemulsion content demonstrated higher moisture contents than those with a moderate quantity of incorporated cinnamon EO nanoemulsion, according to Frank et al. (2018) [84]. However, the variations were negligible, with the moisture content of the films remaining between 10% and 12%. According to Jouki et al. (2014) [92], the rise in moisture content was attributed to the glycerol concentration in the film, while Ghasemlou et al. (2013) [97] proposed that this is due to the hydrophobic character of EOs, which has a direct effect on the water retention of the films.

### 3.3. Mechanical Properties

Tensile strength (TS) and elongation at break (EAB), which are closely related to the chemical structure of edible films, are the most commonly used characteristics to evaluate their mechanical properties [98]. The mechanical attribute of an edible film’s tensile strength reflects the maximum stress that the film can withstand before breaking [82]. Elongation at break, on the other hand, refers to a film’s ability to withstand changes in shape without breaking [75]. A high elongation is desirable for edible films since it improves the film’s capacity to wrap and package food. Table 2 shows the tensile strength and elongation at break of different polysaccharide-based edible films incorporating various EO nanoemulsions. Since water content of polysaccharides-based films varies with the relative humidity of their surrounding atmosphere, it is of utmost importance to pre-equilibrate films in an atmosphere with a relative humidity and a temperature similar to the environmental conditions for their application and to determine its mechanical properties under these conditions. Indeed, the water content of films varies with relative humidity, and water acts as a plastifier of polysaccharide-based films, thereby affecting its mechanical properties.

#### 3.3.1. Tensile Strength

According to Moghimi et al. (2017) [75], there are significant differences in the tensile strength (TS) between control and hydroxypropyl methyl cellulose-based films containing nanoemulsions of *Thymus daenensis* EO. The TS of methyl cellulose edible films when incorporated with *Thymus daenensis* EO nanoemulsions (22.6 ± 0.7 MPa) is lower than the control (36.2 ± 0.7 MPa). When nutmeg EO nanoemulsion was added to a corn starch-based edible film at various concentrations, a similar trend was reported [82]. The observed tensile strengths ranged from 15.1 to 18.9 kgf/mm^2^ and the higher the concentration of nutmeg EO nanoemulsion, the lower the TS of the film.

The TS value was reduced following incorporation of 5 and 7.5% (*v*/*v*) of marjoram EO nanoemulsion in pectin-based edible films [87], which is consistent with the results obtained in previous studies [75,82]. The TS values of pectin-based edible films incorporated with 5 and 7.5% (*v*/*v*) of marjoram EO nanoemulsion obtained from the study were 1.9 MPa and 1.3 MPa, respectively. The decrease in TS could be attributed to the use of EOs in the production of edible films, which makes the films more flexible but reduces their resistance. Because the interaction between polymer and EOs is weaker than the interaction between polymer and polymer, a barrier between hydrocolloid polymer chains and EOs has formed. As a result, in the presence of EOs, the mechanical properties of edible films are compromised [75].

On the other hand, it was reported that incorporation of 3% (*v*/*v*) of *Zataria multiflora* EO nanoemulsion in basil seed gum-based edible films increased the TS value from 19.7 MPa (control) to 34.6 MPa [13]. Furthermore, the TS of methylcellulose-based films increased significantly from 54.2 to 71.7 MPa when incorporated with a plant EO blend (oregano and thyme) nanoemulsion (7.5% *w*/*w*), which is approximately a 30% increase [94]. This phenomenon can be explained by network strengthening caused by extensive hydrogen bondings (dipole-dipole interactions) between polar groups, particularly hydroxyl groups, present along the polymer backbone and polar head groups of surfactant molecules present at the interface of dispersed nanodroplets [13].

Alginate-based edible films incorporated with thyme EO, sage EO, and lemongrass EO nanoemulsions were as resistant as alginate films with no significant differences in TS values [10]. Similarly, the nanoemulsion concentration had no significant effect on TS values, ranging from 3.09 MPa in the edible films without nanoemulsions to 3.43 and 3.23 MPa for edible films with 0.5% (*v*/*v*) of rosemary and lemongrass nanoemulsions, respectively [72]. The reason for this limited variation of TS is most likely due to the low EO nanoemulsion content in the edible films [10].

#### 3.3.2. Elongation at Break

Acevedo-Fani et al. (2015) [10] reported that alginate-based edible films with sage EO nanoemulsions were the most stretchable (78%) when compared to control and other edible films. However, films containing lemongrass EO (32%) and thyme EO (41%) nanoemulsions did not show significant differences compared to the EAB of control films (38%). The influence of the electrical charge of nanoemulsions in the film structure could explain some of the variations in film flexibility. In the case of a charged polymeric film structure, repulsive forces between molecules of the same charge can increase the distance between polymers, resulting in a plasticizing effect [99].

The EAB of basil seed gum composite films increased when incorporated with *Zataria multiflora* EO nanoemulsion (39.5% compared to 21.6% for the control film without *Zataria multiflora* EO nanoemulsion) [13]. Furthermore, there was an increase in EAB in edible films when rosemary and lemongrass EO nanoemulsions (0.5% *w*/*w*) were added, with values of 20.45 ± 1.5% and 23.54 ± 0.8%, respectively, when compared to 10.98 ± 1.2% for the control films [72]. This phenomenon could be explained by incorporating EOs, which weaken the intermolecular interactions between polymer chains, resulting in more extensible and flexible films [100]. Furthermore, due to the reduction in droplet size, EO nanoemulsions have a plasticizing effect on edible films, reducing resistance while increasing elongation [13,72]. However, the EAB of methyl cellulose films only increased from 19.3 to 20.0% when incorporated with oregano and thyme EOs blend nanoemulsion [94].

When 30% (*v*/*v*) glycerol concentration was used in the film formulation, corn starch edible films incorporated with nutmeg EO nanoemulsions showed the highest percentage of EAB of 52.8% [82]. This implies that increasing the glycerol concentration tends to increase the film elongation. The addition of glycerol may reduce the intermolecular force of the edible film matrix structure, increase flexibility, and decrease the number of hydrogen bonds, reducing fragility and making it more difficult to break [82].

## 4. In Vitro Antimicrobial Activity

Many antimicrobial agents found in various types of plant EOs show promising results when tested in vitro against various foodborne pathogens and spoilage bacteria. In Acevedo-Fani et al. (2015) [10], the antibacterial activity against *Escherichia coli* of alginate-based edible films incorporated with nanoemulsions of EOs from various plants such as thyme, lemongrass, and sage was determined using TSA-NaCl medium as a model of a solid food system. Pure alginate edible films exhibited no antimicrobial activity, and microorganism growth was comparable to that of TSA-NaCl plates without film (control). The type of EO, on the other hand, had a significant impact on the antibacterial activity of edible films. The inhibitory effect was significantly stronger in alginate-based edible films incorporated with thyme EO nanoemulsions, whereas lemongrass EO and sage EO nanoemulsion films showed no growth reduction. The presence of thymol molecules, which is the major compound in thyme EO, is likely responsible for the strong inhibitory effect reported in alginate-based films incorporated with thyme EO nanoemulsions. This compound can attach to membrane proteins of microbial cells via hydrophobic interactions, affecting membrane permeability [10].

Furthermore, the antimicrobial activity of basil seed gum-based films incorporated with *Zataria multiflora* EO nanoemulsion was evaluated against *Bacillus cereus* and *Escherichia coli* using broth dilution method [13]. Basil seed gum-based control films had no inhibitory activity against both *Bacillus cereus* and *Escherichia coli*, while the antibacterial activity of *Zataria multiflora* EO nanoemulsion-loaded films increased significantly by incorporating nanoemulsion in film forming suspension. On the other hand, the antimicrobial activity of HPMC edible films containing nanoemulsions of *Thymus daenensis* EO against Gram negative bacteria (*Escherichia coli*, *Salmonella typhi*, *Shigella dysenetriae*, *Shigella flexneri*, *Acinetobacter baumannii* and *Klebsiella peneumoniae*) and Gram positive bacteria (*Staphylococcus aureus*, *Staphylococcus epidermidis*, *Bacillus subtilis*, *Enterococcus faecalis*, *Enterococcus faecium* and methicillin resistant *Staphylococcus aureus* (MRSA)) was evaluated by disk diffusion method in Moghimi et al. (2017) [75]. The results showed that HPMC edible films containing *Thymus daenensis* EO nanoemulsions were more effective at inhibiting these bacteria than pure HPMC edible films (control). It has been proposed that the antibacterial mechanism is due to the presence of thymol and carvacrol, which can cause disruption of the outer membrane of these bacteria.

In a study conducted by Aisyah et al. (2018) [82], the antibacterial activity of corn starch-based edible film incorporated with nutmeg EO nanoemulsion against *Staphylococcus aureus* and *Escherichia coli* was evaluated. The results of the study showed that the edible films with the addition of nutmeg oil nanoemulsion were able to inhibit the growth of these bacteria. In addition, the agar plate containing the edible films containing the highest concentration of nutmeg EO nanoemulsion had the largest inhibition zone against both bacteria. On the other hand, the effect of increasing the concentration of cinnamon EO nanoemulsions on the antimicrobial activity of the SSPS edible film against Gram negative bacteria (*Escherichia coli*, *Pseudomonas aeruginosa* and *Salmonella typhi*) and Gram positive bacteria (*Enterococcus faecium*, *Bacillus cereus* and *Staphylococcus aureus*) was investigated [83]. As expected, SSPS control films revealed no inhibitory effect against both Gram positive and Gram negative bacteria. The SSPS films containing 0.8% cinnamon EO nanoemulsion inhibited both Gram positive and Gram negative bacteria, whereas the 0.4% and 0.6% had no inhibitory effect or only inhibited Gram positive bacteria.

## 5. Food Application of Edible Films Incorporated with Essential Oil Nanoemulsions

Edible films and coatings have gained prominence in recent years due to their advantages over synthetic films, such as their use as active edible packaging in the food industry. The incorporation of specific additives into packaging film or within packaging containers with the purpose of ensuring and extending product shelf-life is referred to as active packaging [101]. Active edible films are preferable to active packaging because the ingredients are only food-grade, and the biopolymers used to produce edible films are commonly derived from food industry waste or underutilized sources of proteins, lipids, or polysaccharides [102]. These active edible film ingredients are biodegradable, edible, and can act as carriers of active agents such as antimicrobials, antioxidants, flavorings, and nutraceuticals [103]. Because of their numerous advantages, these active edible films could be used to preserve fresh and further processed meats, fishes, seafoods, fruits, vegetables, and other foods. Their activity is due to the addition of antioxidants and antimicrobial compounds in edible packaging films, which aid in spoilage prevention, safety, and consumer acceptance [44].

EOs from various plants are commonly incorporated into edible films to produce active edible packaging films. EO-incorporated edible films promote the use of EOs in foods by enhancing their delivery precisely in food areas where pathogens grow and proliferate, as well as enhancing their stability before use [103]. Moreover, by nanoemulsification of EOs, the antibacterial activity of EOs can be enhanced when incorporated into edible films [104]. As a result, EOs nanoemulsion-incorporated edible films are regarded as an effective and innovative method for food quality preservation. Furthermore, EOs nanoemulsion-incorporated edible coatings have additional benefits, such as increasing the biological activity of active substances and thereby reducing the impact on the sensory properties of foods by decreasing their required quantity [104]. Table 3 shows the shelf-life extensions of various food products when packed or coated with different polysaccharide-based edible films incorporating various EO nanoemulsions.

### 5.1. Physico-Chemical Analysis

#### 5.1.1. Colour

The colour of a food product is an important factor in determining its freshness, and it also influences consumer acceptance. In the study conducted by Xiong et al. (2020) [79], oregano EO and resveratrol nanoemulsion-loaded pectin edible films were applied on pork loin and stored at 4 °C for up to 20 days. L*, a* and b* parameters were determined with a colorimeter, and they correspond to lightness, the position between red (positive values) and green (negative values), and are positioned between yellow (positive values) and blue (negative values), respectively. The L* (lightness) values of all groups gradually increased over time, and the L* values of the control samples were significantly higher on day 20 than the L* values of samples coated with oregano EO and reseveratrol nanoemulsion-loaded pectin edible films. This showed that as meat deteriorates, pork becomes paler over time, but coatings with oregano EO and resveratrol could effectively prevent this. Other authors [85] coated Beluga sturgeon fillets with jujube gum-nettle EO nanoemulsion films before storage at 4 °C for 15 days. Similarly to Xiong et al. (2020) [79], the L* values of all samples also increased with storage time. This increase of L* value over storage time might be due to both enzymatic and non-enzymatic reactions that result in the degradation of myofibrillar proteins and the disorganization of myofibrils. However, on day 15, the L* values of the control samples were higher than those of the samples coated with jujube gum edible films with nettle EO nanoemulsion [105].

Besides L* values, the a* parameter allows monitoring the redness of meat, which is a significant aspect for customers to evaluate its freshness. The redness of pork loins increased between 0 and five days, reaching a maximum on day five, and then decreasing during the fresh pork loin preservation under high oxygen modified atmosphere packaging (HOMAP) [79]. The early increase in redness is primarily due to myoglobin pigments being exposed to the high amount of oxygen in a HOMAP, where deoxymyoglobin (purple colour) is converted to oxymyoglobin (bright red colour). However, the deoxymyoglobin and oxymyoglobin pigments oxidize over time to form the brown pigment metmyoglobin, resulting in a reduction in redness and the meat becoming unappealing [106].

Colour and appearance characteristics are also thought to be one of the most influential factors in the marketability of any fruit. Tomatoes were treated with sodium alginate-based edible coatings containing nanoemulsion of *Citrus sinensis* EO and stored at 25 °C and 60% RH for 15 days [107]. During the 15 days incubation period, a* values changed from a light red reading to a red colour. Interestingly, the a* values (red colour) for the untreated controls were significantly lower than those for the treated controls. Donsi et al. (2015) [108] investigated green beans preservation following application of a chitosan-based coating containing nanoemulsion of mandarin EO. In this case, the bioactive coating had no significant effect on the colour of green beans, with all the different colour components following the same trend observed for control samples during refrigerated storage.

**Table 3:** Quality attributes and shelf life of various food products coated with different polysaccharide-based edible films incorporated with nanoemulsified EOs.

Edible Film with Nanoemulsified Essential Oil	Food Product	Shelf Life (Days)	Storage Improvements	References
Citrus peel pectin film with oregano EO and resveratrol	Pork loin	15	- Delayed the process by which pork becomes paler over time - Slowed down the pH reduction in pork - Delayed lipid oxidation in pork - Decreased bacterial activity on the pork	[79]
Chitosan film with *Ferulago angulata* EO	Rainbow trout fillets	16	- Kept TBARS levels below the maximum acceptable level during storage - Delayed the rate of TVC increment during storage - Outperformed the control in terms of quality attribute scores from panelists	[81]
Pullulan with cinnamon EO	Strawberries	6	- Completely inhibited yeast and mould growth during storage	[80]
Soluble soybean polysaccharide with cinnamon EO	Fresh beef meat	8	- Remained the natural pH of meat during eight days of storage - Completely inhibited yeast and mould growth during storage	[83]
Jujube gum film with nettle EO	Beluga sturgeon fillets	15	- Slowed down the pH reduction in Beluga sturgeon fillets - Outperformed the control in terms of quality attribute scores from panelists	[85]
Chitosan film with cumin EO	Beef loin	14	- Kept lipid oxidation in beef loin under control - Outperformed the control in terms of quality attribute scores from panelists	[86]
Pectin film with orange peel EO	Fresh-cut orange	17	- Reduced lactic acid bacteria growth - Inhibited bacterial growth in fresh-cut orange - Reduced yeast and mould growth	[89]
Sodium alginate film with *Citrus sinensis* EO	Tomatoes	7	- Minimized acid break-up	[107]

#### 5.1.2. pH

The determination of pH is important in evaluating the quality of fresh meat. According to Warner et al. (1997) [109], the normal range of good quality fresh pork pH is around 5.4–5.7. From day 20, the uncoated pork had the lowest pH among all the treated pork samples, followed by the pectin-coated sample, and then the oregano EO and/or resveratrol-incorporated coating [79]. This suggests that a pork sample without any thin film protection is susceptible to bacterial spoilage, and that the addition of oregano EO and resveratrol can further improve meat preservation.

Initial pH values of beef meat varied from 5.52 to 5.7 [83]. These pH values slightly increased during storage, with the greatest increase associated with uncoated and coated without cinnamon EO nanoemulsions samples, which reached 6.6 and 6.5 on the eighth day of storage, respectively. The pH of the meat treated with cinnamon EO nanoemulsions-loaded coatings remained in the initial pH range of meat during eight days of storage, suggesting thus that the cinnamon EO nanoemulsion addition in coating significantly reduced the production of basic compounds by food-spoiling microorganisms. Indeed, the increase in pH of control bovine meat and meat coated without cinnamon EO was likely caused by the accumulation of volatile bases such as ammonia and trimethylamine as a result of amino acid degradation by bacterial or enzymatic activities [85].

Jujube gum-nettle EO nanoemulsion film was applied on Beluga sturgeon fillets to extend their shelf-life [85]. The initial pH value of the untreated sample was higher than that of the samples coated with the various concentrations of nettle EO nanoemulsion, and it rapidly decreased until the end of the 15 days refrigerated storage period. The glycogenolysis occurrence or the anaerobic production of lactic acid from glycogen can cause a decrease in pH in fish within the first hours following death [110]. However, a decreasing trend in the pH values of samples coated with nettle EO nanoemulsion was only observed during the first 10 days: pH increase after 10 days is attributed to the build-up of volatile bases such as ammonia and trimethylamine [85]. In addition, increasing the jujube gum content resulted in a decrease in pH value. Because carboxylic acid is a weak acid, this decrease was most likely caused by the accumulation of carboxylic groups of uronic acid forming esters by esterification in the jujube gum structure [111].

Orange peel EO microemulsion and nanoemulsion were added to pectin-based coatings to extend the shelf life of fresh-cut orange [89]. Interestingly, these authors prepared coatings based on citrus industry by-products: indeed, citrus pectin can be extracted from orange juice pomace and orange peel is a by-product of cut fruits. During the storage period, the pH of all samples increased significantly. The uncoated samples had the lowest values in pH, whereas a significant rise in pH was observed in samples coated with a nanoemulsion containing 1% orange peel EO. The lower pH values in the control might be due to an increase in lactic acid bacteria [112]. In the study by Das et al. (2020) [107], after 15 days of storage, the pH of uncoated tomatoes was 4.20, while the pH of tomatoes coated with the sodium alginate-based edible coating containing nanoemulsion of *Citrus sinensis* EO was 3.92. The increase in pH value in control could be due to acid break-up during storage [113].

#### 5.1.3. Thiobarbituric Acid Reactive Substances (TBARS)

The TBARS value is frequently used to assess meat lipid oxidation by measuring second stage auto-oxidation products, mainly aldehydes including malondialdehyde (MDA) [79]. The detectable level of TBARS in food as an objectionable odour has been recommended to be around 1–2 mg MDA eq/kg [114]. The fresh pork samples had an initial TBARS value of 0.12 mg MDA/kg [79]. The TBARS value in the uncoated pork sample increased dramatically and surpassed the threshold on day five, reaching a TBARS value of 4.2 mg MDA/kg on day 20. The lipid oxidation of the samples with oregano EO and resveratrol-incorporated coating was significantly delayed, and their TBARS values were significantly lower when compared to the uncoated control samples. This could be attributed both to the oxygen barrier properties of the pectin coating and to the presence of oregano EO and reseveratrol, which are potent antioxidants known for their ability to scavenge free radicals and interrupt oxidation chain reactions, thereby preventing lipid oxidation [115].

According to Raeisi et al. (2015) [116], the maximum acceptable level without affecting quality in rainbow trout is 5 mg MDA eq/kg. Rainbow trout fillets were coated with chitosan edible films loaded with *Ferulago angulata* EO nanoemulsion and were packed in polyethylene bags and subsequently stored at 4 °C for 16 days [81]. TBARS levels increased in all samples until day four and then demonstrated a constant or decreasing trend until the eighth day, depending on the type of treatment. The decrease in TBARS between days four and eight could be attributed to the reaction of formed malonaldehyde with proteins, amino acids, and glycogen in flesh, which results in a decrease in its amount [117]. TBARS increased in all groups from the eighth day until the end of the storage period. The increase in TBARS suggests that the oxidation of unsaturated fatty acids in fish flesh is proceeding [81]. However, TBARS levels of all samples, including the uncoated trout fillets, remained below the maximum acceptable level during the 16 days storage period.

Chitosan-based edible films incorporated with cumin EO-loaded nanoemulsion were also developed to improve microbiological safety and the quality of beef loins during refrigerated storage [86]. Besides coating with chitosan-based films, beef loins were also subjected to a gamma-irradiation treatment to decrease their initial microbial load. TBARS levels increased in all groups during storage. As expected, due to radiation-induced lipid peroxidation, the TBARS level was significantly higher (*p* < 0.050) in the irradiated groups (gamma-irradiation at 2.5 kGy) compared to the other groups. However, it appears that the combination of chitosan edible film and cumin EO-loaded nanoemulsion was effective in controlling lipid oxidation in beef loin samples since no significant difference in TBARS levels was found between all the treated groups (chitosan edible film loaded with cumin EO nanoemulsion and a combination of these treatments with gamma-irradiation at 2.5 kGy) at the end of the storage [86]. It has been suggested that the reduction in droplet size caused by nanoemulsion formation causes an increase in the specific surface of the nanoemulsified EO, resulting in fast and efficient radical scavenging and thus decreased oxidation processes [118].

### 5.2. Microbiological Analysis

#### Total Viable Count (TVC) and Total Yeast and Mould Count

The coating of pork loin samples had a significant impact on the TVC during storage in a high oxygen modified atmosphere packaging [79]. The initial TVC of fresh pork meat was 3.66 log CFU/g, which was below the threshold of 7 log CFU/g for the acceptable freshness of pork meat [119]. During storage, the TVC value of uncoated pork rose dramatically, surpassing this threshold on day 15, and reaching 9.09 log CFU/g on day 20. During the 20 days of storage, however, all other coated samples were below this threshold. Interestingly, during storage, the sample coated with oregano EO and reseveratrol nanoemulsion pectin edible film had the lowest TVC value [79]. The incorporation of oregano EO or resveratrol in the coating drastically decreased bacterial activity on the pork meat samples, which could be due to their protection in the biopolymer (pectin) matrix, their progressive release on the superficial zone of pork meat and the elevated solubility of oregano EO in the nanoemulsion system due to the reduction in particle size, which improved the coating’s antimicrobial activity [77].

TVC was also monitored during the storage at 4 °C of rainbow trout fillets coated with chitosan edible films loaded with *Ferulago angulata* EO nanoemulsion and subsequently packed in polyethylene bags. For the control group, the TVC of rainbow trout fillets was 3.95 log CFU/g [81]. In raw fish, the TVC in the control group exceeded the maximum suggested level of 7 log CFU/g [120] by day eight. Interestingly, immediately after treatment, the TVC in rainbow trout fillets coated with chitosan edible films loaded with *Ferulago angulata* EO nanoemulsion reduced considerably. TVC then began to increase in each group over the period of 16 days of storage, albeit at a slower rate in EO-coated trout fillets. At day 16, EO nanoemulsion coated fillets had the lowest TVC, about 4.65 log CFU/g lower than the control fillets. It was concluded that at the 8th, 12th, and 16th days of storage, the increase in TVC of fillets treated with chitosan edible films loaded with *Ferulago angulata* EO nanoemulsion was significantly lower than that of fillets treated with conventional chitosan films with *Ferulago angulata* EO conventional emulsion or EO-free chitosan coating [81]. This phenomenon can be explained by the nanoemulsion’s reduced particle size, which results in higher surface-to-volume ratio of EO droplets, allowing antimicrobial compounds to penetrate the bacterial cell membrane more easily and at a faster rate, improving the EO’s antimicrobial effectiveness [121].

Fresh orange slices were coated with pectin and a nanoemulsion of orange peel EO to extend their shelf life [89]. The initial TVC value in fresh orange slices was found to be 3.29 log CFU/g. The TVC of control non-coated and coated orange slices gradually and noticeably rose with storage time. After 17 days of storage, the bacterial load of control orange slices was 6.73 log CFU/g, while samples coated in a pectin-based coating incorporated with orange peel EO nanoemulsion had a 5.83 log CFU/g TVC. Yeast and mould counts of non-coated fresh orange slices and the same samples covered in a pectin-based coating incorporating nanoemulsified orange peel EO were also evaluated in this study [89]. After 17 days of storage at 4 °C, yeast and mould counts increased from 2.57 log CFU/g to 7.66 log CFU/g in uncoated samples, while they increased less in the nanoemulsion-coated samples containing 1% orange peel EO, with 2.78 and 6.93 log CFU/g at days 0 and 17, respectively. It has been concluded that bacterial counts and yeasts and mould counts were lower in samples treated with pectin-based edible coatings containing orange peel EO nanoemulsion than in those without orange peel EO (controls) [89]. The larger amount of orange peel EO released via nanoemulsion may improve the EO’s bioavailability and thereby enhance its antibacterial effects [122]. This is due to the small size of EO droplets in nanoemulsion systems, which ensures a close and wide contact with the surface of the bacteria cell walls, potentially increasing the amount of EO penetrating into the cells and leading to bacterial cellular breakdown [123].

Chu et al. (2020) [80] reported that coating strawberries with a pullulan-cinnamon EO nanoemulsion effectively inhibited yeast and mould growth. Indeed, uncoated strawberries and strawberries coated with pullulan without cinnamon EO had yeast and mould counts over 4 log CFU/g after six days of storage at room temperature, while the yeast and mould count of strawberries coated with pullulan film with cinnamon EO nanoemulsion was only 1.96 log CFU/g. It was deduced that the coatings containing cinnamon EO nanoemulsion maintained effective mould inhibitory activities throughout the storage period [80]. The high antimicrobial activity of cinnamon EO in nanoparticles scattered in the matrix contributed significantly to the antifungal activity of the pullulan-cinnamon EO nanoemulsion coating [124]. This is due to the smaller size and uniform distribution of cinnamon EO droplets in the nanoemulsion in coating, which could maximize the surface area, making antimicrobial compounds in cinnamon EO more accessible to transfer from coatings to food matrices and contact with bacteria [125,126].

Ghani et al. (2018) [83] monitored yeast and mould count during refrigerated storage of meat (control), meat coated with soluble soybean polysaccharides-based films or the same films incorporated with a cinnamon EO micro- or nano-emulsion. Yeast and mould count increased during storage in uncoated meat samples, as well as in samples coated in soluble soybean polysaccharides-based films without cinnamon EO, unlike in samples coated with the same films incorporated with cinnamon EO nanoemulsion or microemulsion. Compared to uncoated samples, the use of these active films resulted in a 1 log cycle decrease in yeast and mould count on day eight. This is most likely due to the inhibitory action of trans-cinnamaldehyde (the main cinnamon EO constituent) on various enzymes such as fungal cell wall synthesizing enzymes [127,128]. Interestingly, there were significant differences between samples coated with films incorporated with microemulsified and nanoemulsified cinnamon EO: incorporation of nanoemulsified EO resulted in a higher antifungal activity [83]. This is consistent with the conclusions of several other authors, who observed that EO nanoemulsions can increase their antimicrobial activity and are highly effective in inactivating microbes [129,130].

### 5.3. Sensory Analysis

Fresh-like quality is an important aspect in terms of consumer satisfaction, which is attributed to the colour, texture, taste, and aroma properties of the product [131]. Uncoated rainbow trout fillets samples received the lowest scores for the quality attribute scores (texture, odour, colour, and overall acceptance) by panellists throughout the entire storage time, except at day 0, followed by samples coated with chitosan alone, samples coated by chitosan incorporated with *Ferulago angulata* EO, and finally samples coated with chitosan incorporated with *Ferulago angulata* EO nanoemulsion, respectively [81]. Interestingly, samples coated with chitosan incorporated with *Ferulago angulata* EO nanoemulsion received scores above the consumers acceptability limit even after 16 days of storage. These results indicate an extension of the shelf life of rainbow trout fillets from eight days, for uncoated samples, to up to 16 days when coated with chitosan films incorporated with nanoemulsified orange peel EO.

Similarly, Gharibzahedi et al. (2017) [85] reported that the panellists’ scores in terms of sensory attributes (odour, colour, taste, tenderness and overall acceptance) of Beluga sturgeon fillets decreased significantly with the increase in storage time, with samples coated with jujube gum incorporated with nettle EO nanoemulsions showing a slower reduction. The delivery systems composed of 3.5% (*w*/*w*) nettle EO nanoemulsions and of 8 or 12% (*w*/*w*) jujube gum received the highest sensory scores from panelists, indicating that they were the most suitable coatings for Beluga sturgeon fillet preservation. High amounts of jujube gum appear to efficiently encapsulate nettle EO nanoemulsions, resulting in increased antibacterial and antioxidant activity. The results thus showed that nettle EO nanoemulsions contribute significantly to the extension of the shelf-life of Beluga sturgeon fillets by delaying the process of deterioration, while providing consumers with a pleasant flavour and/or odour.

The results of sensory evaluations (colour, odour, texture, and overall acceptability) conducted on beef loins received from panelists revealed that the beef loins were well accepted on day 0 [86]. In addition, panelists reported a slight pleasant cumin odour in beef loin samples coated with chitosan edible film based incorporated with cumin EO nanoemulsion at the start of the storage period. Because irradiation can cause unpleasant odours such as burnt, bloody, sweet, sulphur, or pungent odours [132], it has been explained that the pleasant odour of the cumin EO nanoemulsion masked the potential unpleasant irradiation odours in samples which were irradiated before being coated. During the storage period, all sensory quality scores declined considerably in all experimental groups, albeit at a slower rate in the samples coated with chitosan films incorporated with nanoemulsified cumin EO than in the uncoated samples. Based on sensory scores, chitosan edible film combined with cumin EO nanoemulsion and low-dose gamma irradiation was thus the most effective treatment for maintaining the sensory attributes of loins during storage. This latter study is a good example of the interest in combining the use of polysaccharides-based coatings/films incorporated with nanoemulsified EOs with other factors inactivating the growth of food-spoiling microorganisms to extend the shelf life of perishable foods, consistently with hurdle technology principles.

## 6. Conclusions and Perspectives

The rising consumer demand for natural food preservation in recent years has prompted the development of mild preservation technology which will improve the product’s quality and safety without causing nutritional and sensory loss. The use of EOs nanoemulsions instead of synthetic food preservatives such as potassium sorbate, sulphites, or nitrites is in line with the “clean label” trend, which is defined as the reformulation of foods with fewer or no preservatives. Moreover, the utilization of EOs nanoemulsions in edible films has opened up a new field of study for the development of edible films for food preservation. This paper discussed the various types of EO nanoemulsions used in the development of active edible films, the physical, chemical, mechanical, and antimicrobial properties, as well as the potential of applications for the preservation of various types of perishable foods of these EO nanoemulsion-containing edible films. Extensive research on the volatility of EO nanoemulsions, as well as the release kinetics of nanoemulsified EO constituents once incorporated into edible films, is required to provide a better understanding of how EOs act after being incorporated as nanoemulsions into active edible films.

## Figures and Tables

**Table 1:** Formulation of different polysaccharide-based edible films incorporating various EO nanoemulsions.

Polysaccharides	Solvents	Plasticizers	Surfactant (*v*/*v*)	Essential Oil Nanoemulsions	References
Sodium alginate	Distilled water	Glycerol (2% *v*/*v*)	Tween^®^ 80 (3% *v*/*v*)	*Thymus vulgaris* *Cymbopogon citratus* *Salvia officinalis*	[10]
Hydroxyl propyl methyl cellulose	Distilled water	Polyethylene glycol (0.01, 0.1, 0.5% *w*/*v*)	Tween^®^ 80 (10% *w*/*w*)	*Thymus daenensis*	[75]
Basil seed gum	Double distilled water	Glycerol (1.5% *w*/*v*)	Tween^®^ 80 (4.5% *w*/*w*)	*Zataria multiflora*	[13]
Corn starch	Distilled water	Glycerol (10%, 20%, 30% *v*/*v*)	Tween^®^ 80 (20% *w*/*v*)	*Myristica fragrans*	[82]
Citrus peel pectin	Milli-Q water	-	Tween^®^ 80 (5% *v*/*v*)	*Origanum vulgare*	[79]
Chitosan	Acetic acid	-	Tween^®^ 80 (1, 2, 3% *v*/*v*)	*Ferulago angulata*	[81]
Pullulan	Distilled water	Glycerol (15% *w*/*w*)	Tween^®^ 80 (2% *w*/*w*)	*Cinnamomum verum*	[80]
Soluble soybean polysaccharide	Distilled water	Glycerol (50% *w*/*w*)	Soy protein isolate (1% *w*/*v*) Lecithin (0.05% *w*/*v*)	*Cinnamomum verum*	[83]
Alginate	Distilled water	Glycerol (20% *w*/*w*)	Tween^®^ 80 (1, 2, 3, 4, 5% *v*/*v*)	*Cinnamomum verum*	[84]
Banana starch	Distilled water	Glycerol (1% *w*/*v*)	Tween^®^ 80 (0.5–2 *w*/*w*)	*Cymbopogon citratus* *Salvia rosmarinus*	[72]
Jujube gum	Buffer acetate solution	Glycerol (15% *w*/*v*)	Tween^®^ 40 (20% *w*/*v*)	*Urtica dioica*	[85]
Chitosan	Glacial acetic acid	Glycerol (25% *w*/*w*)	Tween^®^ 80 (6% *v*/*v*)	*Cuminum cyminum*	[86]
Citrus pectin	Distilled water	Glycerol (20% *w*/*w*)	Tween^®^ 80 (30% *w*/*w*)	*Origanum majorana* Pickering	[87]
Methyl cellulose Cellulose nanocrystal	Distilled water	Glycerol (0.13% *w*/*w*)	Tween^®^ 80 (-) Lecithin (-)	Blended (*Origanum vulgare* and *Thymus vulgaris*)	[88]
Pectin	Double distilled water	-	Tween^®^ 80 (4% *v*/*v*)	*Citrus sinensis* peel	[89]

**Table 2:** Tensile strength and elongation at break values of different polysaccharide-based edible films incorporating various EO nanoemulsions.

Edible Film	Essential Oil Nanoemulsion	Tensile Strength (MPa)	Elongation at Break (%)	References
hydroxypropyl methyl cellulose	*Thymus daenensis*	22.6	14.2	[75]
corn starch	*Myristica fragrans*	15.8	32.9	[82]
pectin	*Origanum majorana*	1.3	21.1	[87]
basil seed gum	*Zataria multiflora*	34.6	39.5	[13]
methyl cellulose	*Origanum vulgare* *Thymus vulgaris*	71.6	20.0	[94]
banana starch	*Cymbopogon citratus* *Salvia rosmarinus*	3.2 3.4	23.5 20.5	[72]
